# Development of High-Titer Antidrug Antibodies in a Phase 1b/2a Infant Clesrovimab Trial Are Associated With RSV Exposure Beyond Day 150

**DOI:** 10.1093/infdis/jiae582

**Published:** 2024-11-26

**Authors:** Nithya Thambi, Jia Yao Phuah, Ryan P Staupe, Lori M Tobias, Yu Cao, Troy McKelvey, Radha A Railkar, Antonios O Aliprantis, Carmen Sofia Arriola, Brian M Maas, Kalpit A Vora

**Affiliations:** Department of Epidemiology; Department of Epidemiology; Department of Epidemiology; Department of Infectious Diseases and Vaccines; Department of Pharmacokinetics Dynamics Metabolism Bioanalytics; Department of Infectious Diseases and Vaccines; Department of Pharmacokinetics Dynamics Metabolism Bioanalytics; Department of Pharmacokinetics Dynamics Metabolism Bioanalytics; Department of Infectious Diseases and Vaccines; Department of Biostatistics and Research Decision Sciences; Department of Clinical Research Translational Medicine; Department of Epidemiology; Department of Infectious Diseases and Vaccines; Department of Quantitative Pharmacology & Pharmacometrics, Merck & Co., Inc., Rahway, New Jersey, USA; Department of Epidemiology

**Keywords:** RSV, clesrovimab, passive immunization, antidrug antibodies (ADA), serum neutralizing antibodies (SNA)

## Abstract

**Background:**

Clesrovimab is a human half-life–extended monoclonal antibody in phase 3 evaluation for the prevention of respiratory syncytial virus (RSV) disease in infants. Antidrug antibodies (ADA) were observed at late time points in a phase 1b/2a study where clesrovimab was well tolerated with an extended half-life of approximately 45 days.

**Methods:**

Serum samples at days 150, 365, and 545 postdose were assayed for ADA titers. Samples with high ADA titers were characterized for their binding specificity to the Fab or the YTE portion of clesrovimab. RSV serum neutralization (SNA) titers were also measured on ADA-positive and ADA-negative infants. Additionally, a D25 (site Ø) competitive enzyme-linked immunosorbent assay (ELISA) was performed on ADA-positive available samples to determine RSV exposure. Local surveillance data was used to ascertain RSV circulation during the trial.

**Results:**

High ADA titers were observed in a minority of infants at days 365 and 545 for all doses tested. Additionally, all high-titer ADA-positive infants had ADA directed towards the YTE epitope of clesrovimab. Moreover, these infants demonstrated robust RSV-SNA and had D25 competitive antibodies suggesting an RSV exposure after day 150, coinciding with the epidemiological data.

**Conclusions:**

RSV exposure in infants beyond day 150 after dosing is associated with ADA development and high RSV-SNA titers with no impact on pharmacokinetics.

**Clinical Trials Registration:**

NCT03524118.

## Teaser

RSV exposure in clesrovimab-treated infants, beyond the intended clesrovimab therapeutic window, is associated with the development of high-titer antidrug antibodies and concomitant higher levels of respiratory syncytial virus neutralizing antibodies. Clinical Trial Registration. NCT03524118

Respiratory syncytial virus (RSV) infects the respiratory epithelium resulting in bronchiolitis (inflammation of the small airways in the lung) and pneumonia in children less than 1 year of age. RSV is the leading cause of lower respiratory tract infection-related deaths during the neonatal period [1]. While the severity of RSV infection can vary, it can lead to significant respiratory distress in infants and is the most common cause of hospitalizations within the first 6 months of life [2]. Recently, significant progress has been made to address RSV infection, with the emergence of new vaccines and monoclonal antibodies (mAbs) as active and passive immunizations, respectively. All approved modalities target the prefusion conformation of the RSV fusion (F) protein, a glycoprotein that is crucial for viral entry into the host cell and the primary target for potent neutralizing antibodies. Amongst mAbs, palivizumab (targeting site II) and nirsevimab (targeting site Ø) have shown efficacy in preventing RSV disease. Palivizumab is administered monthly during the RSV season for high-risk infants younger than 24 months [3]. Clesrovimab (targeting site IV) is a mAb derived from the fully human parental antibody RB1, with the YTE half-life modification to the Fc region [4] under investigation in phase 3 for efficacy against RSV disease in infants. A single dose of clesrovimab at the start of the RSV season is expected to provide protection against RSV disease in infants for the full season (approximately 150 days). Nirsevimab is also a half-life–extended antibody that was recently approved as a single dose given intramuscularly for RSV prophylaxis in infants [5].

While mAbs have revolutionized the field of biologic therapies, their immunogenicity leading to the development of antidrug antibodies (ADA) poses a challenge. ADA responses arise when treated individuals mount a humoral immune response against the mAb, typically targeting either the Fab or the Fc portion. ADAs can potentially lead to reduced efficacy due to faster drug clearance or may block the action of mAbs by acting as neutralizing antibodies, leading to a loss of therapeutic effect [6]. Moreover, ADA can lead to rare adverse immune reactions locally or systemically, due to type III hypersensitivity. These reactions have been infrequently observed in children below the age of 10 years as compared to adults. The onset of type III hypersensitivity is within 7–10 days after repeat administration of the drug and self-resolves with drug discontinuation and clearance [7, 8]. The effects of ADA were mostly learned from early biologics such as tumor necrosis factor (TNF) inhibitors in the context of autoimmune disease [9]. Therefore, understanding immunogenicity and ADAs in RSV mAb therapies is crucial for development.

In a recent phase 1b/2a trial, 36.7% of the clesrovimab treated infants developed ADA in the per-protocol population. These ADA did not alter the half-life of clesrovimab [10]. Interestingly, a disproportionate number of ADA-positive infants manifested high RSV serum neutralization titers at day 365 and day 545, leading us to hypothesize that RSV exposure after day 150 was associated with the ADA response. The majority of high RSV-serum neutralizing antibodies (SNA) titer infants also displayed D25 competitive (site Ø) antibodies confirming RSV exposure between days 150 and 545. These findings were further correlated with RSV circulation in the geographical regions the infants were enrolled at, in support of RSV exposure. Taken together, these data support a model wherein RSV exposure at time points beyond the window of intended RSV prophylaxis of clesrovimab (ie, within 150 days of dosing) can drive an ADA response, which is not anticipated to impact pharmacokinetics (PK).

## METHODS

### Study Design

Healthy male and female preterm and full-term infants were administered clesrovimab in a randomized, phase 1b/2a, dose-finding trial, as described in Madhi et al [10]. Data from a total of 53 infants with ADA-positive status were analyzed (Supplementary Figures 1 and 2). These were composed of the 51 ADA-positive participants reported in Madhi et al [10] from the per protocol population, as well as 2 additional participants with blood samples that were obtained outside of the protocol-specified window.

### Measurement of Serum Levels of Clesrovimab

Serum clesrovimab concentration was quantified using liquid chromatography-tandem mass spectrometry as previously described [11, 12] with a lower limit of detection at 0.5 µg/mL as detailed in Madhi et al [10].

### Measurement of Antidrug Antibodies

A previously described electrochemiluminescence immunoassay [11] was used to assess ADA status within sera of participants as reported by Madhi et al [10]. Sera samples from clesrovimab-dosed cohorts at baseline, day 150, and 365 were used for ADA assessment. Furthermore, ADA assessment was also carried out for samples at day 545 only for participants in the 100-mg dose group. High ADA titers are defined as ≥1000 and low ADA titers are defined as ≤200.

### Measurement of RSV Serum Neutralizing Antibodies

RSV-SNA titers were measured using a virus reduction neutralization assay as previously described [13]. Sera RSV-SNA titer data at baseline, day 150, and day 365 from infants in the 20, 50, 75, and 100-mg dose groups was used in this study. RSV-SNA from day 545 was measured only from the 100-mg dose group.

### Serum Antibody Epitope Mapping/Binding Characterization

ADA-positive infant samples were characterized for epitope specificity using an electrochemiluminescent (ECL) assay on the Meso Scale Discovery (MSD) platform. Biotin-labeled and sulfo-tag–labeled clesrovimab, together with motavizumab-YTE [14] or RB1 (parental mAb to clesrovimab), as competing antibodies, were incubated with serum samples enabling the formation of complexes with anti-clesrovimab antibodies present in the sample. Precomplexed samples were added to streptavidin-coated MSD plates and read using an MSD plate reader. The ECL signal is proportional to the concentration of anti-clesrovimab antibodies in the samples.

### D25 Competition Assay

RSV D25 competition assay is an AlphaLISA immunoassay that detects antibodies competing with the anti-prefusion RSV F antibody D25 for binding sites on the RSV prefusion F protein. The assay was performed as described previously [15].

### RSV Epidemiology Data

Local RSV surveillance data [16–19], reported as RSV positivity rate, was overlayed with matched trial duration (from treatment to day 365) of each ADA-positive infant at their respective locations.

## RESULTS

### Antidrug Antibodies Targeting Clesrovimab Primarily Target the YTE Epitope

In a recent phase 1b/2a clinical trial, 139 of the 143 infants dosed with clesrovimab had samples for ADA assessment. Of these, 19 infants were ADA positive of 127 (15%) evaluated at day 150. Twenty-two infants were ADA positive of 85 (26%) evaluated at day 365. Finally, 30 infants were ADA positive from 47 (64%) infants evaluated in the ADA assay at day 545 (Supplementary Figure 3). Overall, 53 of 139 evaluated clesrovimab-treated infants, developed ADA at some point during the study (Supplementary Figures 1 and 2). However, ADA status had no apparent impact on the PK of clesrovimab when compared to infants without an ADA response, as reported in Madhi et al [10].

**Figure 1.**
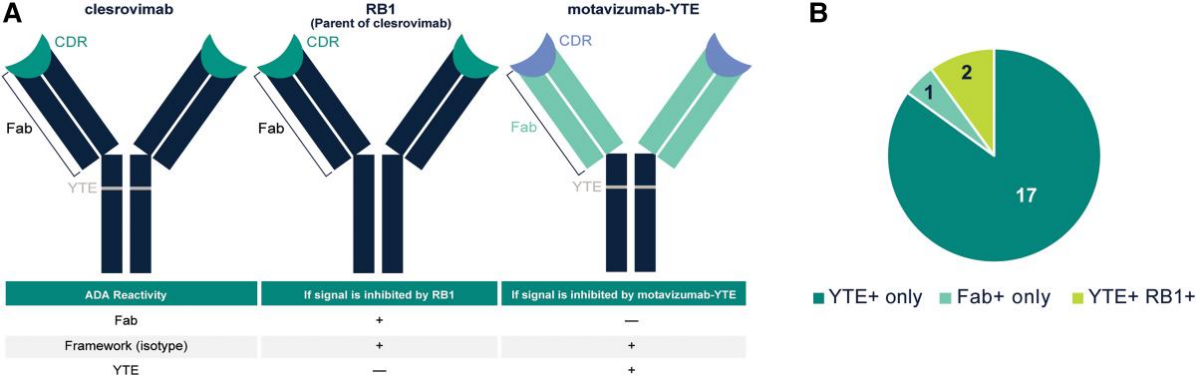
Antidrug antibodies (ADA) targeting clesrovimab primarily target the YTE domain. *A*, Schematic describing the ADA characterization assay: ADA-positive infant sera were incubated with clesrovimab and either of the competing antibodies, RB1 (parental mAb to clesrovimab without YTE) and motavizumab engineered with YTE. Inhibition of clesrovimab binding in the presence of RB1, but not motavizumab-YTE would suggest that tested serum had ADA directed to the Fab portion of clesrovimab. Alternatively, inhibition of binding to clesrovimab in the presence of motavizumab-YTE but not RB1 would signify a YTE-specific ADA response. *B*, The chart shows data summarizing ADA specificity with 17/20 infants showing specificity to YTE, 1/20 infant to Fab portion, and 2/20 specific to both YTE and Fab portion of clesrovimab.

To better understand ADA in clesrovimab-treated infants, we sought to characterize antidrug reactivity of serum ADA responses in ADA-positive individuals. Clesrovimab is a fully human antibody containing the half-life extension YTE mutations in the Fc domain [4]. We hypothesized that the YTE epitope could be the primary target of ADA in clesrovimab-treated patients, as previously reported for nirsevimab [20]. To test this, we developed a qualitative, competitive binding assay to distinguish whether ADAs were targeted against the Fab or the YTE epitope in the Fc region of clesrovimab (Figure 1*A*). Using this assay, we tested samples from infants that had high ADA titers (days 150, 365, and 545 time points) for their ADA specificity. Of the 20 infants with available samples, 17 scored positive for YTE specificity and 2 infants had positivity for both YTE and Fab of clesrovimab at days 365 and/or 545, and 1 infant had positivity at day 150 for Fab specificity (Figure 1*B*).

### Serum Neutralizing Activity Is High in ADA-Positive Infants

**Figure 2.**
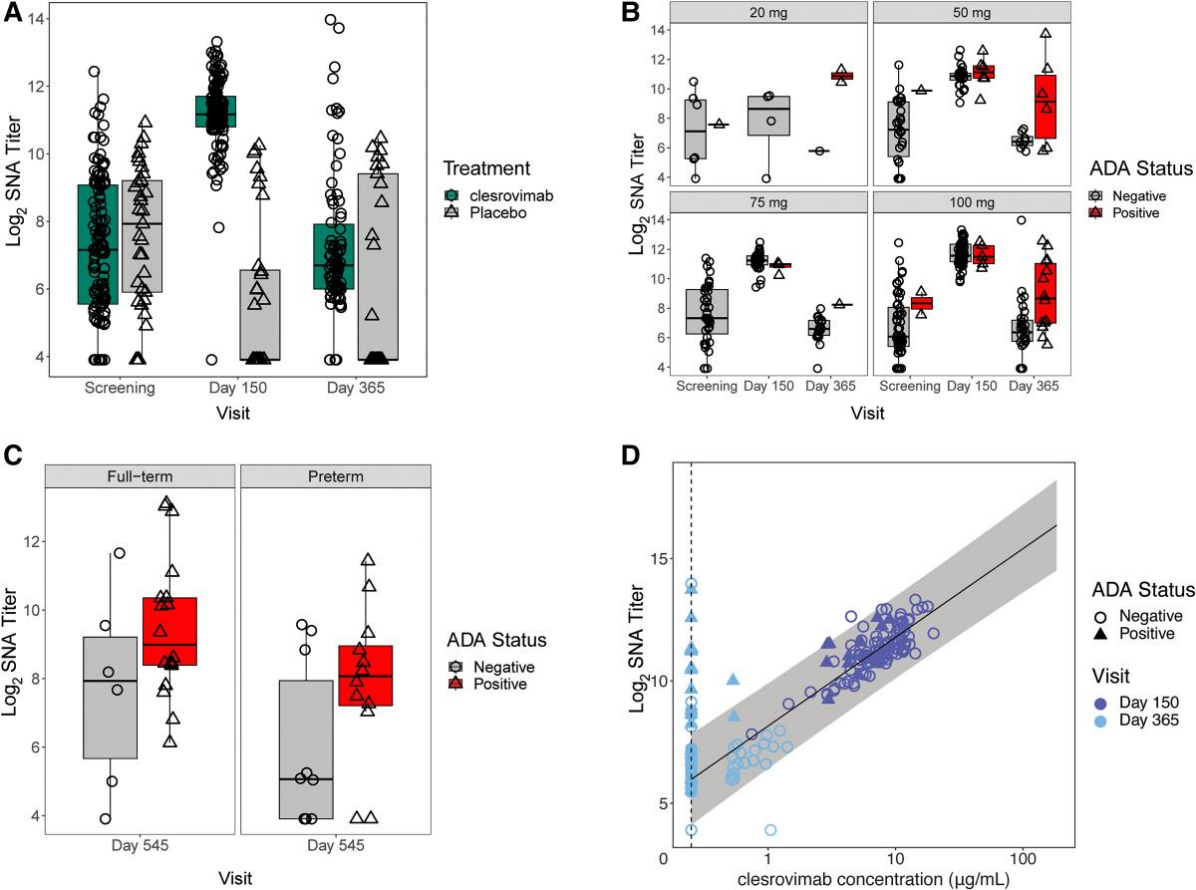
SNA is higher in ADA-positive infants. *A*, RSV-SNA titers in clesrovimab (open circles) and placebo (open triangles) treated infants at screening, day 150, and day 365 time points. *B*, RSV-SNA titers in ADA-positive (open triangles) and ADA-negative (open circles) infants at screening, day 150, and day 365 time points, stratified by individual dose groups. *C*, RSV-SNA titers in ADA-positive (open triangles) and ADA-negative (open circles) infants at day 545 time point, stratified by pre- and full-term infants. The box is bounded by the 25th and 75th percentiles; the line within the box represents the median. The upper whisker extends from the upper hinge to the largest value no further than 1.5 × IQR from the hinge. The lower whisker extends from the lower hinge to the smallest value at most 1.5 × IQR of the hinge. *D*, Each point represents a paired SNA titer and serum clesrovimab concentration, of either ADA-positive (empty circles) or ADA-negative (closed triangles) infants at day 150 (dark blue) and day 365 (light blue) time points. Black line represents the fitted relationship between SNA titers and serum clesrovimab concentration. The shaded gray area represents the 99% prediction interval of the above fitted relationship. Abbreviations: ADA, antidrug antibodies; IQR, interquartile range; RSV, respiratory syncytial virus; SNA, serum neutralizing antibodies.

We next sought to understand whether development of ADA responses impacted pharmacodynamics (PD) in clesrovimab-treated infants. RSV-SNA was observed at screening for most infants in both the placebo and clesrovimab-treated groups, likely resulting from maternal transfer (Figure 2*A*). RSV-SNA were high in clesrovimab-treated infants at day 150, reflecting the PK concentrations, as shown in Madhi et al [10], because serum concentrations of clesrovimab are directly correlated with RSV-SNA [21, 22]. Furthermore, at this time point RSV-SNA were lower in the majority of placebo-treated infants due to decay of maternal antibodies (ie, a substantial proportion of placebo-treated infants had titers below the lower limit of detection). At day 365, RSV-SNA titers remained low in the placebo group, but interestingly were elevated in infants who received clesrovimab, compared to placebo (Figure 2*A*), despite there being little to no drug in circulation at this time point (Figure 2*D*). Further analysis of the individual clesrovimab dose groups revealed that ADA-positive infants had higher RSV-SNA titers at day 365 (approximately 4–8 fold; Figure 2*B*) and day 545 (Figure 2*C*) than the ADA-negative infants. It is important to note that day 545 samples were only collected for the 100-mg dose group and the finding was similar in pre- and full-term infants (Figure 2*C*). This difference in the RSV-SNA titers with ADA status was not apparent at day 150.

Another way of representing the data is shown in Figure 2*D*, wherein the data are plotted for clesrovimab concentrations against RSV-SNA titers. The line represents the expected relationship between concentration of clesrovimab and RSV-SNA. The shaded regions define the 99% prediction interval for this relationship. All day 150 samples but 1, and the majority of samples from ADA-negative infants at day 365, fall within the expected intervals for the relationship of drug concentrations to RSV-SNA titers (Figure 2*D*). However, ADA-positive infants at day 365 do not adhere to this relationship and show a high RSV-SNA titer despite having low to undetectable clesrovimab concentrations, likely indicating RSV exposure. Because infants were enrolled in this trial prior or during the start of their first RSV season, days 365 and 545 likely fell into the second RSV season.

### ADA-Positive Infants With High SNA Likely Experienced RSV Exposure Beyond Day 150

**Figure 3.**
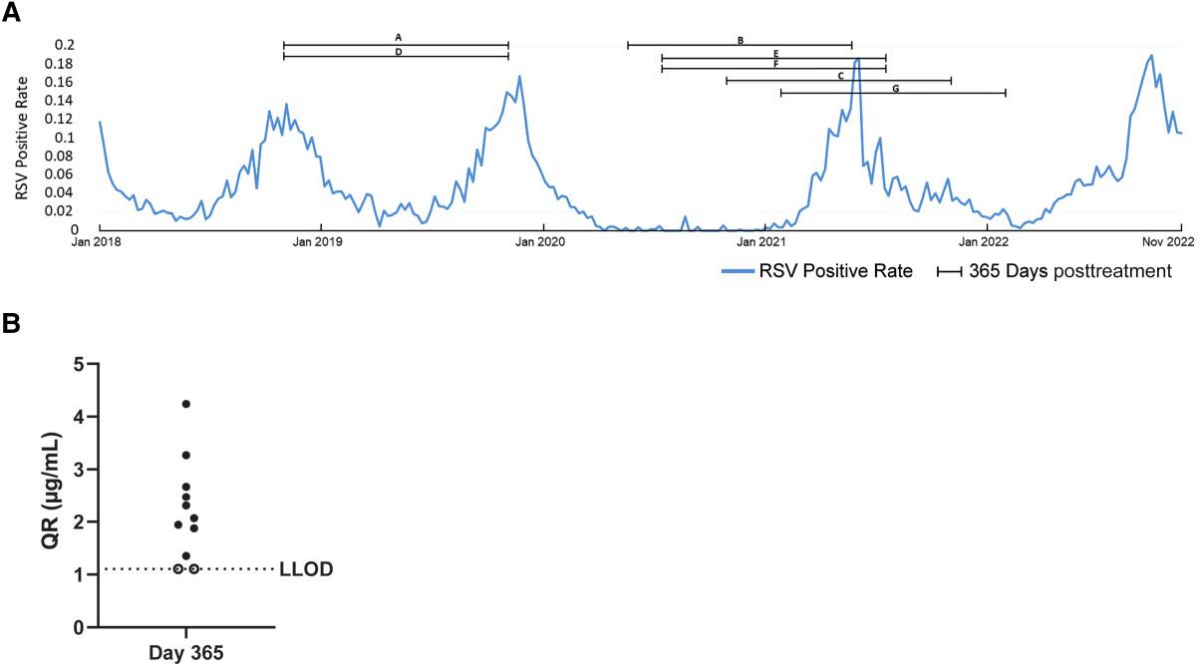
ADA-positive infants with high SNA likely experienced second-season RSV exposure. *A*, Representation of RSV-positive cases in Florida, United States across time with overlay of dosing timelines until day 365 for infants A–G located at that clinical trial site. *B*, D25 (RSV site Ø) antibody competition at day 365. Nine of 11 infant sera (≥1000 ADA titers, closed circles) showed > LLOD signal in the AlphaLISA assay, detecting site Ø antibodies at day 365. Two of 11 infant sera that were below the LLOD (open circles) were imputed at LLOD value of 1.105 µg/mL for graphing purposes. Abbreviations: ADA, antidrug antibodies; LLOD, lower limit of detection; QR, quantifiable range; RSV, respiratory syncytial virus; SNA, serum neutralizing antibodies.

We reasoned that RSV exposures at later time points (beyond day 150, coinciding with the second RSV season) could trigger high RSV-SNA and ADA responses. Because the PK for clesrovimab is low at day 365, the higher RSV-SNA activity would suggest a recent exposure to RSV. We investigated the RSV circulation at each clinical trial site during the time of our phase1b/2a clinical trial. We restricted our analyses to only clesrovimab-treated infants, 14 of whom had ADA titer >200 (Supplementary Figure 4). In Figure 3*A* and Table 1, we depict 1 such trial location in Florida, United States and show the dosing timelines until day 365, overlayed with RSV circulation for 7 infants, A–G, from this region. Infants A–F had second-season RSV peak closer to their day 365 sampling time point. In contrast, infant G who had low ADA titers, had RSV exposure roughly 6 months prior to day 365. It is interesting to note that all of the infants with >200 ADA titer had RSV circulating in their geographical regions between day 150 and day 365 (Supplementary Figure 4).

**Table 1.** Infant ADA Titers at 365 Days Posttreatment

Infant	Treatment Start	Titer
A	Nov 2018	134 000
B	Jun 2020	1200
C	Nov 2020	3440
D	Nov 2018	16 000
E	Aug 2020	5220
F	Aug 2020	18 000
G	Feb 2021	147

While our analysis of RSV circulation at each clinical site is aligned with the hypothesis that ADA-positive infants experienced RSV exposure after day 150, we sought a second independent assay to further support this claim. RSV exposure induces host polyclonal antibody responses that target each of the major antigenic sites on RSV prefusion F (pre-F) protein (sites Ø, I, II, III, IV, and V/VIII) [23]. We rationalized that because clesrovimab specifically binds to antigenic site IV on RSV prefusion F protein [4], the presence of serum anti-RSV antibodies targeting nonsite IV epitopes would be caused by viral exposure. Therefore, we used a D25 (antibody that recognizes site Ø on RSV pre-F) competitive binding assay for detecting the presence of antibodies targeting the highly immunogenic site Ø epitope [24]. Of the 11 high-titer infants who had available samples, we observed site Ø targeted serum antibodies at day 365 in 9 infants and 2 that were below lower limit of detection for the assay (Figure 3*B*). These data suggest that most of these infants likely experienced an RSV exposure between days 150 to 365, coinciding with periods of local RSV activity from surveillance data.

**Figure 4.**
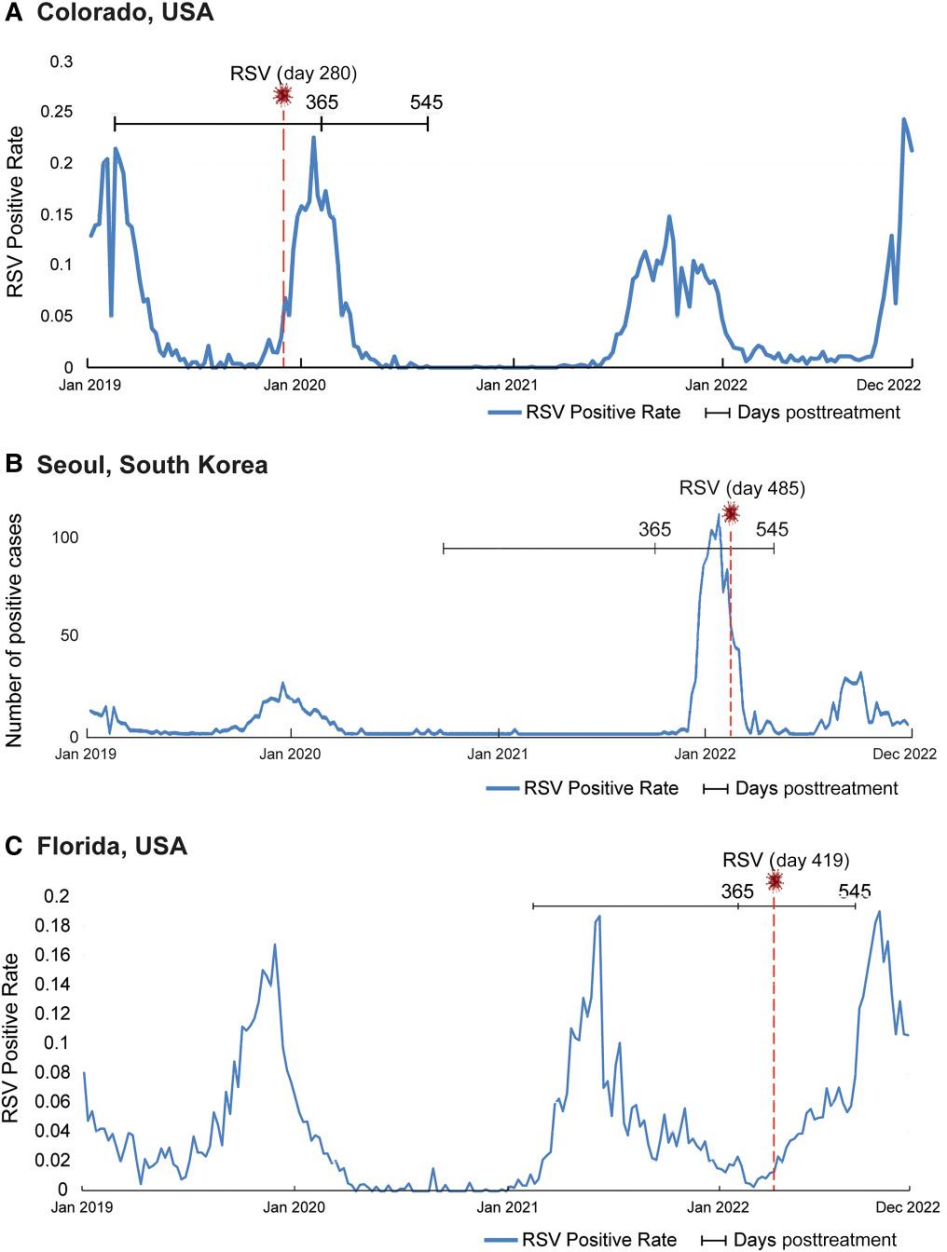
Three clesrovimab-treated infants that were clinically diagnosed with RSV by PCR beyond day 150 are depicted. *A*–*C*, Each graph depicts 1 infant, showing the period of study (solid horizontal line) beginning with clesrovimab administration with time points day 365 and 545 after clesrovimab treatment: (*A*) infant A located in Colorado, United States, diagnosed with RSV at day 280; (*B*) infant B located in Seoul, South Korea, diagnosed with RSV at day 485; and (*C*) infant C located in Florida, United States, diagnosed with RSV at day 419. The RSV diagnosis along the time interval is depicted (vertical dotted line and relative day after clesrovimab administration). The curves depict the local RSV positive rate as reported by the respective public health RSV surveillance agencies. Abbreviations: PCR, polymerase chain reaction; RSV, respiratory syncytial virus.

Additionally, we examined 3 clesrovimab-treated infants that had clinically diagnosed RSV infection by PCR beyond day 150 (Figure 4 and Table 2). Infant A was diagnosed with RSV infection at day 280, coincident with an uptick of RSV circulation in their region. In infant A, ADA titers were observed at day 365, but were negative at day 150. Infants B and C were diagnosed with RSV infection at days 485 and 419, respectively. Consistently, ADA titers for both infants B and C were observed at time points after RSV diagnosis at day 545 and were negative at prior time points. We expected to see D25-specific antibodies and a concomitant rise in SNA titers at time points where ADA positivity was observed. In these cases, high-titer ADA was observed only after the clinically diagnosed RSV infection and corroborates with the hypothesis that RSV exposure led to ADA development in these infants.

**Table 2.** ADA, RSV-SNA, and D25 Competition Titers for Infants A–C at Indicated Time Points

Infant	ADA Titer			SNA Titer				D25 Competition, µg/mL	
	Day 150	Day 365	Day 545	Baseline	Day 150	Day 365	Day 545	Day 365	Day 545
A	Negative	35 300	NS	669	2062	35 513	NS	1.4	NS
B	Negative	Negative	3130	185	8214	<30	1633	NA	2.8
C	NS	Negative	4860	53	NS	53	391	NA	1.4

ADA, RSV-SNA, and D25 competition titers are shown for the 3 infants in Figure 4. Only ADA-positive samples were assayed.

Abbreviations: ADA, antidrug antibodies; NA, not assayed; NS, no sample; RSV, respiratory syncytial virus; SNA, serum neutralizing antibodies.

Because D25 positivity could not be assayed for all samples, there was no way to confirm RSV infection in some infants. To supplement this analysis, we categorized infants into a suspected RSV category, which was defined as having an SNA titer at day 365 above the 99th percentile of the expected SNA value (as determined by the concentration-SNA relationship). This definition of suspected exposure was determined to be reasonable because concentrations of clesrovimab at this time point are negligible and the only source of neutralization titer would be from the host immune system in response to RSV exposure. The incidence of suspected RSV exposure was then compared to the incidence of ADA positivity at the same time point. The probability of being ADA positive at day 365 after likely RSV exposure (as defined above) was 73.7% (14/19) versus 12.7% (8/63) without a likely RSV exposure (Table 3). Of the 8 ADA-positive cases in Table 3 that had low RSV-SNA titers (below the cutoff of 99th percentile for RSV-SNA titers), 5 of the 8 had lower ADA titers (≤1000). Three of the 8, however, do have high ADA titers but their RSV-SNA titers were low. Interestingly, these 3 infants had confirmed site Ø antibodies at both day 365 and day 545. While the presence of RSV F site Ø antibodies suggests an RSV exposure, their lower RSV-SNA titers is confounded by the lack of information on their exact time of RSV exposures due to limited sampling time points.

**Table 3.** Categorization of Infants as Suspected RSV Based on ADA Status and SNA Titers

ADA Status at Day 365	RSV Based on SNA Titer Day 365		
	Yes	No	Total
Positive	14	8^a^	22
Negative	5	55	60
Total	19	63	82

Categorization of infants as “suspected RSV” based on ADA status and SNA titers (above [“yes”] and below [“no”] 99th percentile of expected SNA value, as determined by the concentration-SNA relationship).

Abbreviations: ADA, antidrug antibodies; RSV, respiratory syncytial virus; SNA, serum neutralizing antibodies.

^a^Three infants had high ADA titers, but low SNA titers. Of these, 2 had D25 below lower limit of detection at day 365, but were D25 positive at day 545; very little RSV was circulating between day 365 and day 545 for these 2 subjects. The third subject was D25 positive at both day 365 and day 545. The other five infants had both low ADA titers and low SNA titers.

## DISCUSSION

These experiments were carried out to understand the ADA responses observed in a phase1b/2a clinical trial for clesrovimab in infants. Our analysis found that overall, a minority of infants were ADA positive (53 of 139). The ADA titers were low at day 150 except for a single infant that had high anti-Fab specific ADA titers in the 75-mg dose group (Figure 1*B*). It is important to note that the presence of ADAs at any time point did not impact the PK or PD activity of clesrovimab until day 150. The safety findings in these infants are reported in Madhi et al [10]; ADA status was not associated with any safety concerns including hypersensitivity reactions. SNA titers in clesrovimab-treated infants at day 365 were higher than placebo control (Figure 2*A*). Twenty-six percent of infants dosed with clesrovimab were ADA positive at day 365 and, interestingly, the ADA-positive infants had higher median RSV-SNA (approximately 4–8 fold higher) than ADA-negative infants (Figure 2*B*). Because the amount of clesrovimab in the serum at day 365 cannot account for this high RSV-SNA titer (Figure 2*D*), we hypothesized that infants with high SNA titers at day 365 had experienced an RSV exposure between day 150 and day 365 that then caused the ADA response. We evaluated this hypothesis in infants who had high ADA titers and noted a good correlation between high ADA and SNA titers in most infants (Supplementary Figures 1 and 2). Furthermore, we were able to demonstrate that there was RSV circulation in the geographical areas where these high-titer ADA-positive infants were located, thus supporting the hypothesis that RSV exposure after day 150 leads to high ADA and SNA titers (Figure 3*A* and Supplementary Figure 4). Additionally, at day 365, 9 of the 12 infants (Figure 3*B* and Supplementary Figure 1) with high ADA titer, had D25 competitive antibodies; however, we could not assay 1 infant due to sample unavailability. These data suggest an immune response to RSV site Ø, which is only possible upon exposure to RSV in the second season.

We saw similar trends at day 545 wherein 36.1% (17/47) had high ADA titers (Supplementary Figure 2) and 14 of 17 infants who had samples remaining for additional analysis were positive for site Ø antibodies. The local RSV surveillance data for all 14 infants suggested RSV circulation between days 365 and 545 (data not shown).

The evidence presented in this article indicates that exposure to RSV beyond day 150 and into the second RSV season led to higher RSV-SNA in clesrovimab-treated infants than placebo controls, with a concomitant increase in ADA titers primarily directed to the YTE (foreign) portion of the fully human mAb. Furthermore, this phenomenon occured when the clesrovimab concentration was low to negligible at the time of RSV exposures. These data indicate that subtherapeutic levels of clesrovimab could augment anti-RSV responses upon new exposure to RSV beyond day 150.

There is evidence in the literature that when there is excess mAb to its cognate antigen, the antigen is rapidly cleared (a situation analogous to the first 150–180 days after clesrovimab dosing). However, when the mAb concentration is lower in comparison to antigen concentration, the antigen-antibody immune complex could become immunogenic and subsequently result in development of ADA [25]. This phenomenon was observed early during introduction of anti-TNF biologics, where lower drug concentrations and longer intervals of dosing led to ADA development [26]. Changing the dosing regimen from episodic to regular intervals led to lowering of ADA incidence [27]. Therefore, formation of immune complexes low mAb to antigen ratios was highly immunogenic with loss of tolerance to both the antigen and the drug [26]. Similar observations were reported when the dosing intervals were longer for asthma biologics [28]. In addition, there is evidence that binding of antigen-antibody complex to dendritic cells via Fc domain interaction would enable T cell help to activated B cells and more antibody production and prove beneficial. Immune complexes have also been tested as vaccine antigens to enhance immune responses against viral [29–33] and bacterial antigens [34]. We hypothesize that at time points after administration with lower clesrovimab concentrations and an RSV exposure (between days 180 and 365), an immune response is developed towards both RSV (RSV-SNA titers) and clesrovimab (anti-YTE titers). This hypothesis is also supported by low ADA titers at day 150, where only the ADA high-titer serum was not YTE-specific.

Intriguingly, RSV exposure during the second season could explain discrepant ADA observations in anti-RSV antibody clinical trials for nirsevimab, as their phase 2b trial [35] reported far fewer ADA occurrences when compared to their phase 1b trial [20]. Furthermore, to the best of our knowledge, a nirsevimab phase 3 trial in the northern hemisphere saw a similar trend of lower ADA, which probably could be explained by a disruption of RSV circulation due to the coronavirus disease 2019 (COVID-19) pandemic [36]. However, it is important to note that the observed incidence of ADA is highly dependent on the sensitivity and specificity of the assay. Differences in assay methods make a meaningful comparison of the incidence of ADA between different studies challenging.

The primary objective of the small-cohort phase 1b/2a study was to address the safety and tolerability of clesrovimab in infants. Investigating the mechanism underlying the elevated ADA became an additional focus only after the data had been gathered at the conclusion of the trial. Therefore, no samples were collected for the purpose of ADA characterization. Due to the participants’ age, we were severely restricted in collecting frequent blood samples, leading to several limitations for this study in terms of sample availability. These limitations also impacted our ability to ascertain the exact time of emergence of ADA in infants in this small study. We will further evaluate our hypothesis in 2 ongoing, larger phase 3 studies (NCT04767373 and NCT04938830).

In summary, we hypothesize that when clesrovimab concentrations are above the RSV F antigen amounts, the RSV virus would be cleared prior to infection, as is the case until day 150. Beyond day 150, if an infant is exposed to RSV, the declining clesrovimab concentration is below the concentration of RSV F antigen. This scenario results in anti-RSV and ADA responses. In other words, ADA development is a consequence of RSV exposure beyond day 150.

## Supplementary Data

Supplementary materials are available at *The Journal of Infectious Diseases* online (http://jid.oxfordjournals.org/). Supplementary materials consist of data provided by the author that are published to benefit the reader. The posted materials are not copyedited. The contents of all supplementary data are the sole responsibility of the authors. Questions or messages regarding errors should be addressed to the author.

## Supplementary Material

jiae582_Supplementary_Data

## Notes

***Acknowledgments.*** We thank Kara S. Cox and Debora Rausch for careful reading and critical reviewing of the manuscript, and Michael Pish from Merck Creative Studios for manuscript figure preparations.

***Author contributions*.** A. O. A. and K. A. V. were involved in the conceptualization or design of the study. L. M. T., Y. C., T. M., and C. S. A. participated in the collection or generation of study data. N. T., J. Y. P., R. A. S., R. A. R., A. O. A., B. M. M., and K. A. V. were involved in data analysis or interpretation. All authors reviewed the manuscript and approved the final version for submission.

***Financial support*.** This work was supported by Merck Sharp & Dohme LLC, a subsidiary of Merck & Co, Inc, Rahway, NJ.
