# Supplementary material for: Development of High-Titer Antidrug Antibodies in a Phase 1b/2a Infant Clesrovimab Trial Are Associated With RSV Exposure Beyond Day 150
Source: J Infect Dis. 2024 Nov 26;231(3):e488–96. doi: 10.1093/infdis/jiae582 (PMC11911791; doi:10.1093/infdis/jiae582)
Supplement: jiae582_Supplementary_Data [file jiae582_supplementary_data.zip › JID-80548R1_manuscriptsupplementalfigureswithlegends_revised.pdf]

Supplementary figure 1

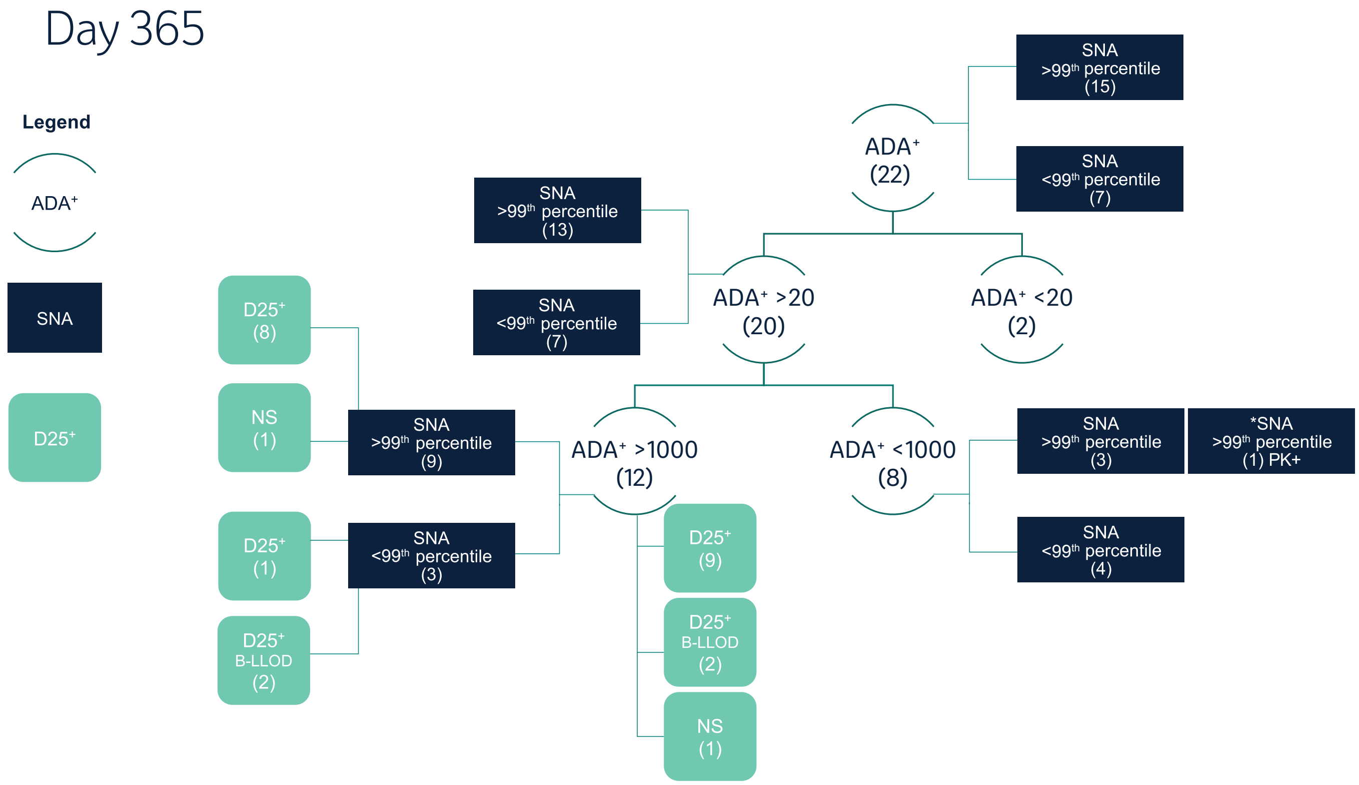

Supplementary figure 1. The flowchart depicts the breakdown of the ADA^{+} infants with samples at day 365 according to quantifiable ADA titers of 20 as the assay detection limit and high or low ADA titers with a cutoff at 1000. SNA titer status is shown where infants with SNA >99th percentile and <99th percentile (dark blue rectangles). A single infant with SNA >99th percentile was notable as the infant still had detectable clesrovimab concentrations in serum (indicated by *). Infants are further subdivided according to D25 assay status (green squares) of positive (D25^{+}), D25 values below lower limit of detection (D25^{+} B-LLOD) or not tested due to absence of sample (NS). D25 assay was only performed on infants with ADA ≥1000. Numbers in brackets denote the number of individual infants in the node.

Supplementary figure 2

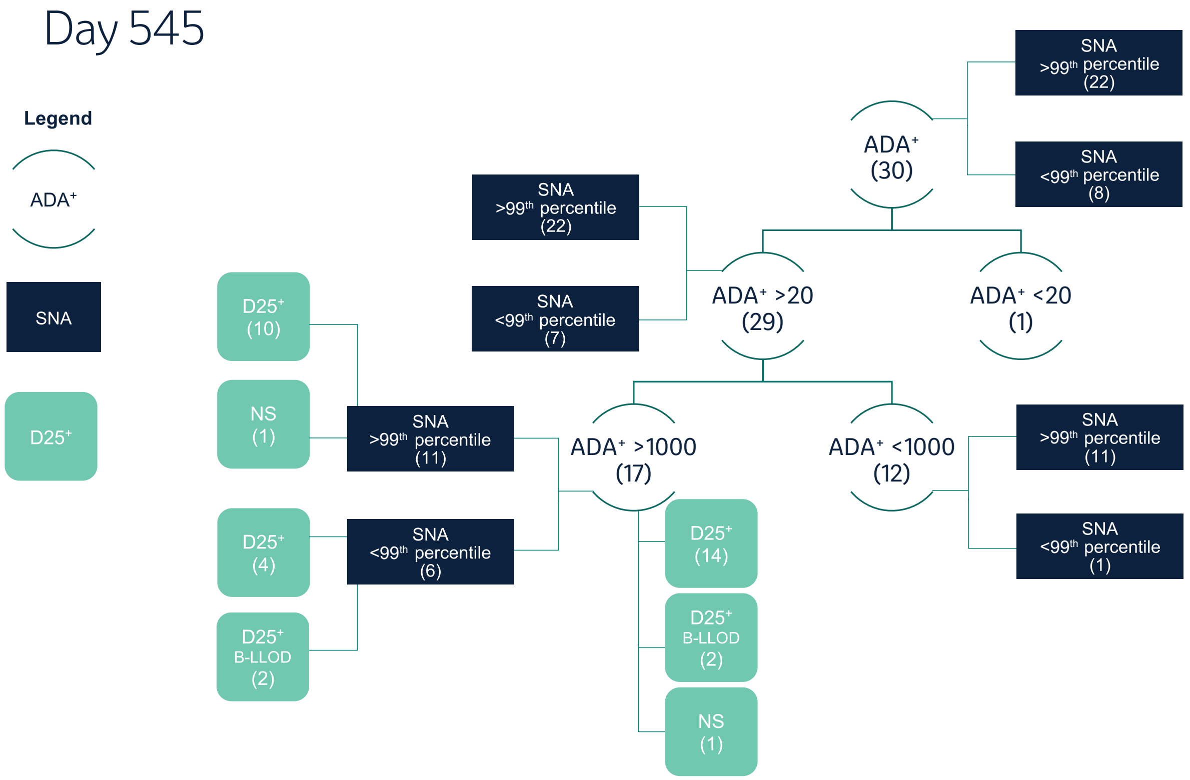

Supplementary figure 2. The flowchart depicts the breakdown of the ADA^{+} infants with samples at day 545 according to quantifiable ADA titers of 20 as the assay detection limit and high or low ADA titers with a cutoff at 1000. SNA titer status is shown where infants with SNA >99th percentile that and <99th percentile (dark blue rectangles). Infants are further subdivided according to D25 assay status (Green squares) of positive (D25^{+}), D25 values below lower limit of detection (D25^{+} B-LLOD) or not tested due to absence of sample (NS). D25 assay was only performed on infants with ADA ≥1000. Numbers in brackets denote the number of individual infants in the node.

Supplementary figure 3

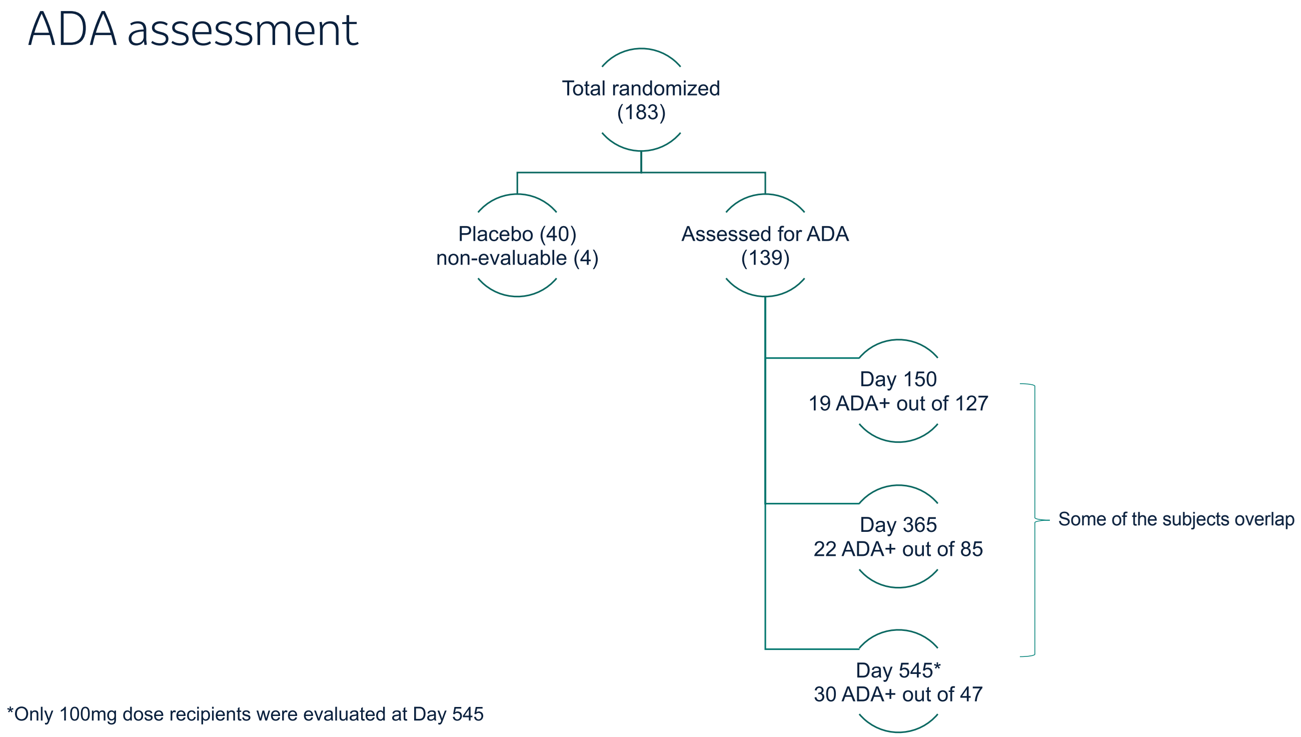

Supplementary figure 3. The flowchart depicts the breakdown of total participants in the Phase 1b/2a study for assessment of ADA status. Of the initial 183 infants, 139 infants in the clesrovimab cohort were assessed for ADA status. ADA status was assessed at time points of day 150 and 365, where ADA+ numbers were reported out of total available samples collected at each time point. Infants in the 100mg dose group had an additional sample collection at day 545.

Supplementary figure 4: Analyses of RSV epidemiology in various clinical sites

A. Chile

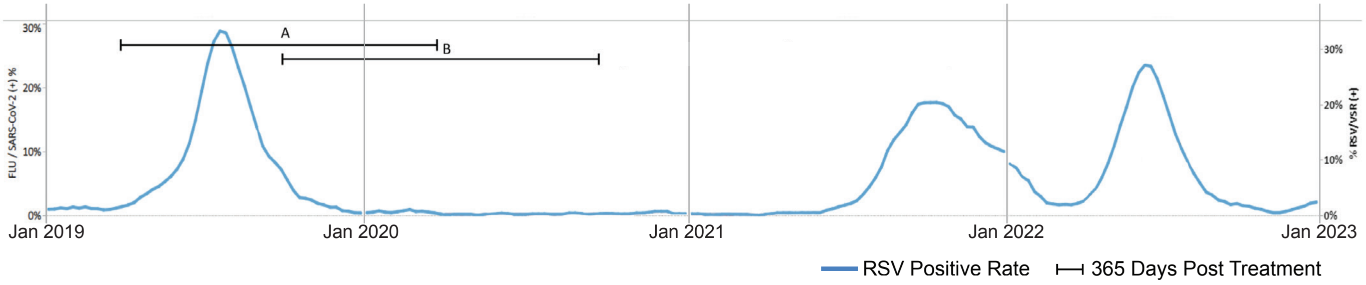

Infant	Treatment Start	ADA positive at 365 days post treatment Titer
A	Apr 2019	73.5
B	Oct 2019	65.5

B. Colombia

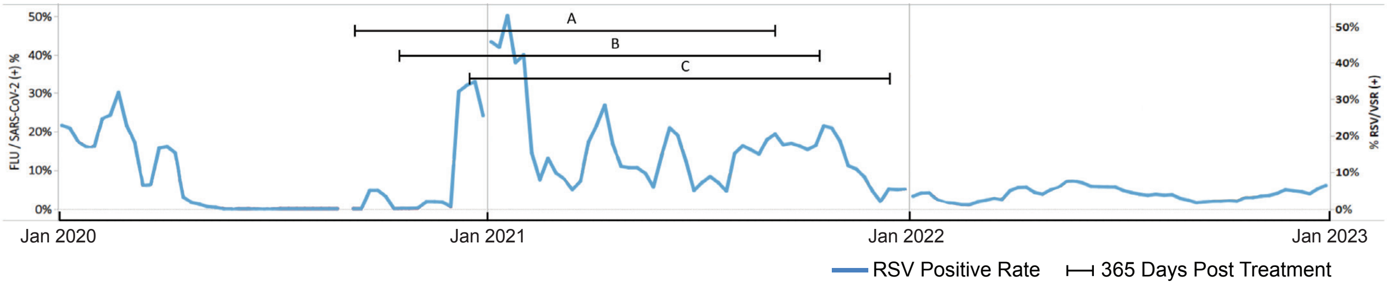

Infant	Treatment Start	ADA positive at 365 days post treatment Titer
A	Sep 2020	1670
B	Oct 2020	2550
C	Dec 2020	676

C. Spain

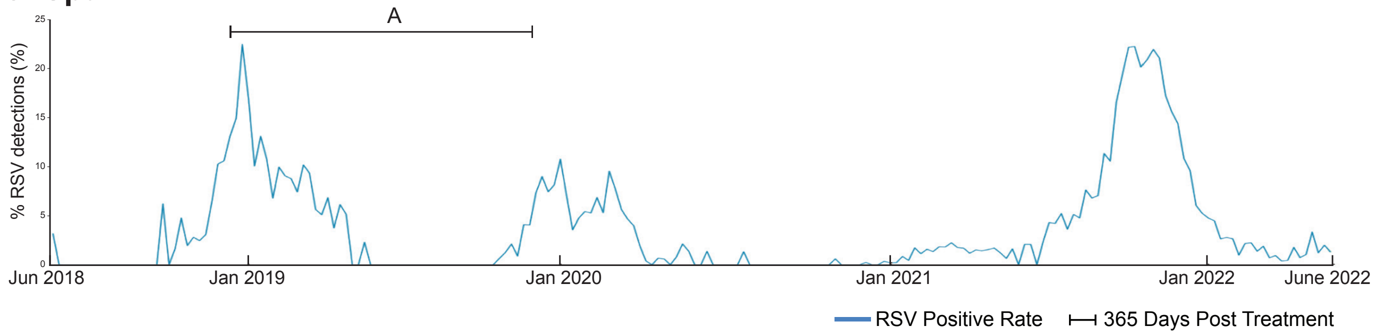

Infant	Treatment Start	ADA positive at 365 days post treatment Titer
A	Dec 2018	10,900

Supplementary figure 4: Analyses of RSV epidemiology in various clinical sites

D. South Africa

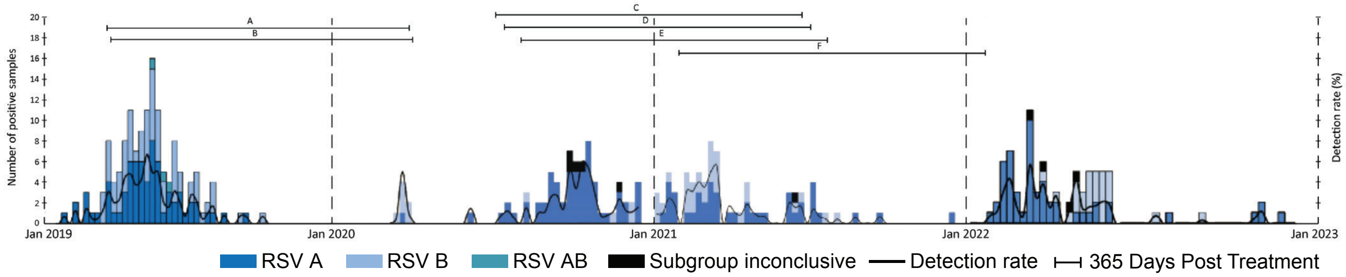

Infant	Treatment Start	ADA positive at 365 days post treatment Titer
A	Mar 2019	59.4
B	Mar 2019	20
C	Jun 2020	126
D	Jul 2020	49.2
E	Jul 2020	121,000
F	Jan 2021	75.4

E. Ohio, USA

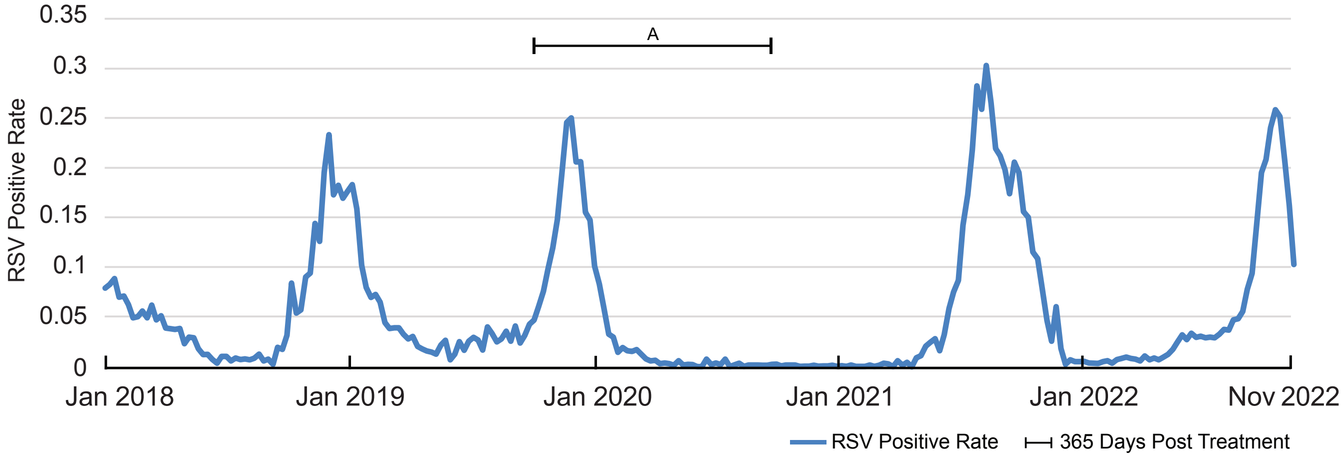

Infant	Treatment Start	ADA positive at 365 days post treatment Titer
A	Sep 2019	20

Supplementary figure 4: Analyses of RSV epidemiology in various clinical sites

F. Wisconsin, USA

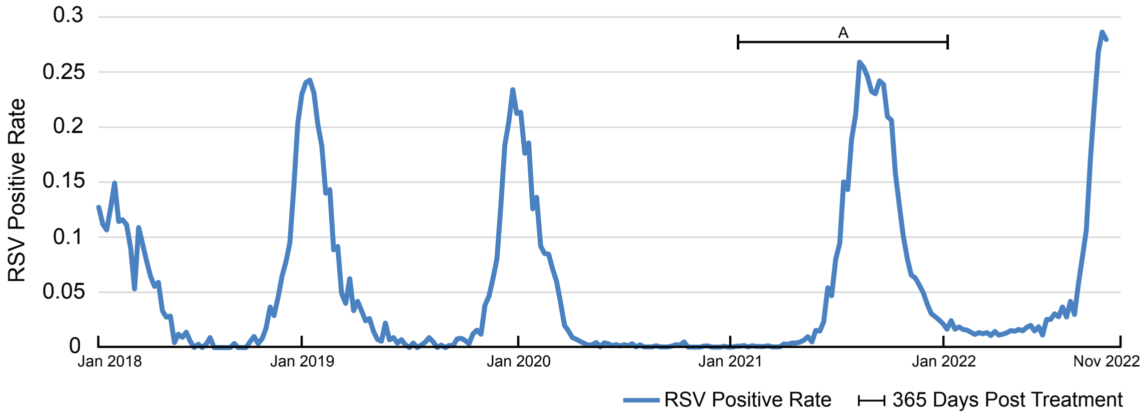

Infant	Treatment Start	ADA positive at 365 days post treatment Titer
A	Jan 2021	2,050

G. Kansas, USA

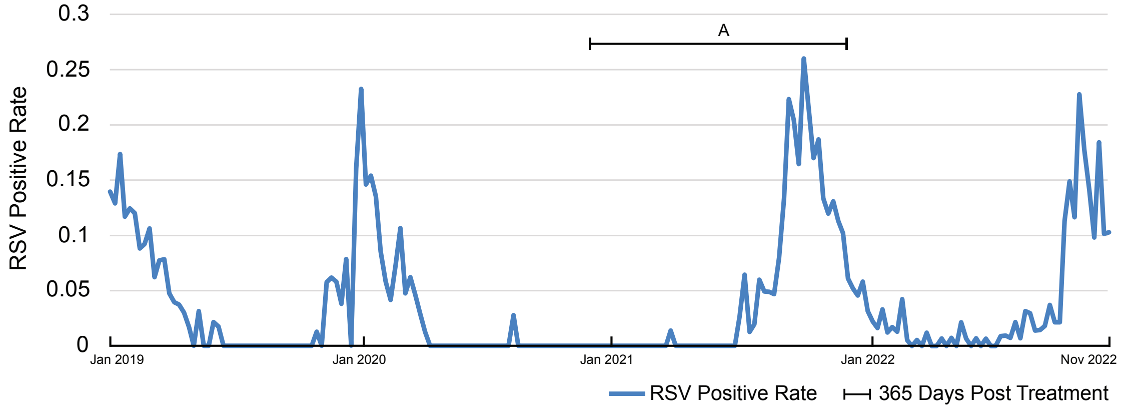

Infant	Treatment Start	ADA positive at 365 days post treatment Titer
A	Nov 2020	724

H. Colorado, USA

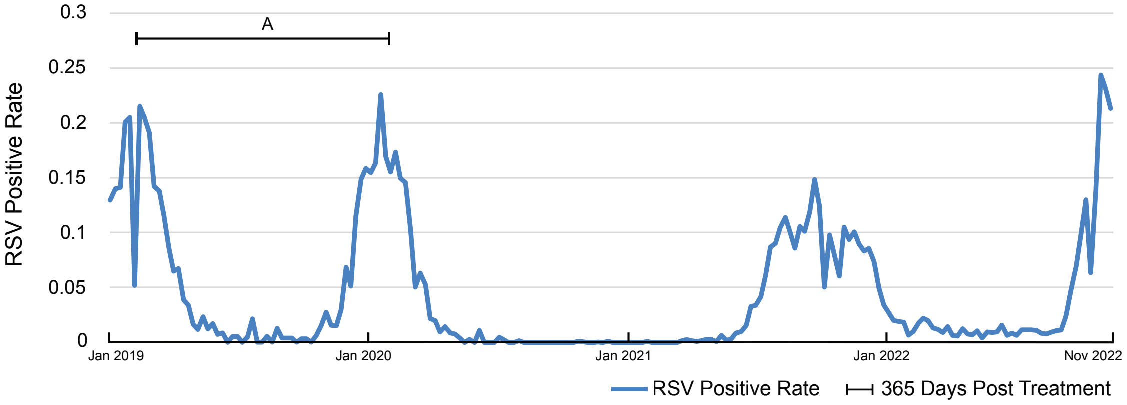

Infant	Treatment Start	ADA positive at 365 days post treatment Titer
A	Feb 2019	35,300

Supplementary figure 4. Each subfigure depicts the RSV detection rate longitudinally (Blue line) at the respective study sites. RSV epidemiology data was adapted using the following sources for stated locations, PAHO for Columbia and Chile, NICD weekly respiratory pathogens database for South Africa, European CDC Infectious Disease Surveillance Atlas for Spain, and CDC for US regions. The study period of each ADA^{+} infant from treatment with clesrovimab to day 365 (black horizontal line) is overlayed at each study site. The table summarizes the approximate treatment start date and ADA+ titer values for each infant associated with the respective study location. A) RSV data from Chile. B) RSV data from Columbia. C) RSV data from Spain. D) RSV data from South Africa. E) RSV data from Ohio, USA. F) RSV data from Wisconsin, USA. G) RSV data from Kansas, USA. H) RSV data from Colorado, USA.
