# Supplementary material for: Development of High-Titer Antidrug Antibodies in a Phase 1b/2a Infant Clesrovimab Trial Are Associated With RSV Exposure Beyond Day 150
Source: J Infect Dis. 2024 Nov 26;231(3):e488–96. doi: 10.1093/infdis/jiae582 (PMC11911791; doi:10.1093/infdis/jiae582)
Supplement: jiae582_Supplementary_Data [file jiae582_supplementary_data.zip › JID-80548R1_supplementalfigurelegends_revised.docx]

**Supplementary Data Legends**

**Supplementary figure 1.** The flowchart depicts the breakdown of the ADA^+^ infants with samples at day 365 according to quantifiable ADA titers of 20 as the assay detection limit and high or low ADA titers with a cutoff at 1000. SNA titer status is shown where infants with SNA > 99^th^ percentile represents high SNA and <99^th^ percentile being low (Dark blue circles). A single infant with SNA > 99^th^ percentile was notable as the infant still had detectable clesrovimab concentrations in serum (indicated by *). Infants are further subdivided according to D25 assay status (green squares) of positive (D25^+^), D25 values below lower limit of detection (D25^+^ B-LLOD) or not tested due to absence of sample (NS). D25 assay was only performed on infants with ADA ≥1000. Numbers in brackets denote the number of individual infants in the node.

**Supplementary figure 2.** The flowchart depicts the breakdown of the ADA^+^ infants with samples at day 545 according to quantifiable ADA titers of 20 as the assay detection limit and high or low ADA titers with a cutoff at 1000. SNA titer status is shown where infants with SNA >99^th^ percentile that represents high SNA and < 99^th^ percentile being low (Dark blue circles). Infants are further subdivided according to D25 assay status (Green squares) of positive (D25^+^), D25 values below lower limit of detection (D25^+^ B-LLOD) or not tested due to absence of sample (NS). D25 assay was only performed on infants with ADA ≥1000. Numbers in brackets denote the number of individual infants in the node.

**Supplementary figure 3.** The flowchart depicts the breakdown of total participants in the Phase 1b/2a study for assessment of ADA status. Of the initial 183 infants, 139 infants in the clesrovimab cohort were assessed for ADA status. ADA status was assessed at time points of day 150 and 365, where ADA^+^ numbers were reported out of total available samples collected at each time point. Infants in the 100mg dose group had an additional sample collection at day 545.

**Supplementary figure 4.** Each subfigure depicts the RSV detection rate longitudinally (Blue line) at the respective study sites. RSV epidemiology data was adapted using the following sources for stated locations, PAHO for Columbia and Chile, NICD weekly respiratory pathogens database for South Africa, European CDC Infectious Disease Surveillance Atlas for Spain, and CDC for US regions. The study period of each ADA^+^ infant from treatment with clesrovimab to day 365 (black horizontal line) is overlayed at each study site. The table summarizes the approximate treatment start date and ADA^+^ titer values for each infant associated with the respective study location. A) RSV data from Chile. B) RSV data from Columbia. C) RSV data from Spain. D) RSV data from South Africa. E) RSV data from Ohio, USA. F) RSV data from Wisconsin, USA. G) RSV data from Kansas, USA. H) RSV data from Colorado, USA.
